# Toward an application of automatic evaluation system for central facial palsy using two simple evaluation indices in emergency medicine

**DOI:** 10.1038/s41598-024-53815-5

**Published:** 2024-02-10

**Authors:** Naoki Ikezawa, Takayuki Okamoto, Yoichi Yoshida, Satoru Kurihara, Nozomi Takahashi, Taka-aki Nakada, Hideaki Haneishi

**Affiliations:** 1 Graduate School of Science and Engineering, Chiba University, Chiba, Japan; 2 Center for Frontier Medical Engineering, Chiba University, Chiba, Japan; 3 Department of Neurological Surgery, Chiba University Graduate School of Medicine, Chiba, Japan; 4 Department of Neurosurgery, Narita Red Cross Hospital, Chiba, Japan; 5 Department of Emergency and Critical Care Medicine, Chiba University Graduate School of Medicine, Chiba, Japan

**Keywords:** Ambulance service, Automatic evaluation system, Emergency medicine, Evaluation index, Facial palsy, Biomedical engineering, Stroke

## Abstract

A stroke is a medical emergency and thus requires immediate treatment. Paramedics should accurately assess suspected stroke patients and promptly transport them to a hospital with stroke care facilities; however, current assessment procedures rely on subjective visual assessment. We aim to develop an automatic evaluation system for central facial palsy (CFP) that uses RGB cameras installed in an ambulance. This paper presents two evaluation indices, namely the symmetry of mouth movement and the difference in mouth shape, respectively, extracted from video frames. These evaluation indices allow us to quantitatively evaluate the degree of facial palsy. A classification model based on these indices can discriminate patients with CFP. The results of experiments using our dataset show that the values of the two evaluation indices are significantly different between healthy subjects and CFP patients. Furthermore, our classification model achieved an area under the curve of 0.847. This study demonstrates that the proposed automatic evaluation system has great potential for quantitatively assessing CFP patients based on two evaluation indices.

## Introduction

A stroke, the second leading cause of death worldwide and a major cause of disability^1^, is a neurological deficit that results mainly from an acute focal injury of the central nervous system due to vascular causes, including cerebral infarction, intracerebral hemorrhage, and subarachnoid hemorrhage^2^. It is a medical emergency and thus requires immediate treatment. Paramedics—the first healthcare contact for most stroke patients^3^—must accurately assess suspected stroke patients and promptly transport them to a hospital with stroke care facilities^4–6^.

To accurately identify suspected stroke patients, the Cincinnati Prehospital Stroke Scale (CPSS) is widely used for emergency medical services^7^. The CPSS assesses the presence of facial palsy, asymmetric arm weakness, and speech abnormalities in suspected stroke patients^8^. Although the CPSS has excellent reproducibility among paramedics and physicians, the reproducibility of the assessment of facial palsy is the worst of the three items of the CPSS^8^. Paramedics and physicians typically instruct patients to smile or show their teeth to assess whether both corners of the mouth symmetrically move when assessing facial palsy with the CPSS; however, this assessment method relies on such a subjective visual assessment, leading to the potential for uncertain assessment.

**Figure 1:**
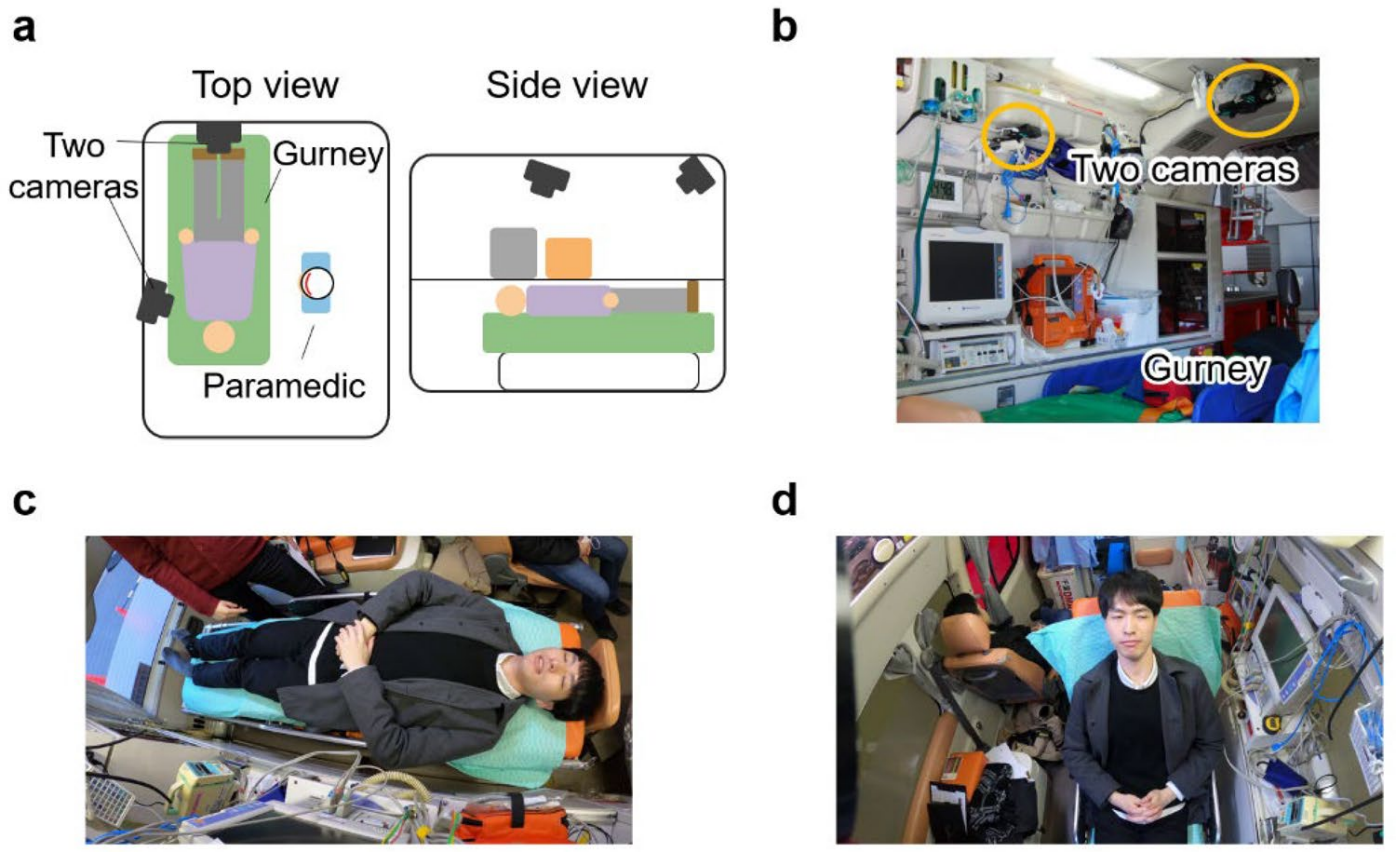
Camera arrangement inside an ambulance for the proposed system. **a** Conceptual image. **b** Photograph of actual environment. Images captured by two cameras with **c** flat gurney and **d** raised gurney.

This study aims to develop an automatic evaluation system for central facial palsy (CFP), one of whose major causes is brain diseases such as stroke, that can be installed in an ambulance. We have been developing a system that allows us to evaluate CFP patients based on video frames of the patient taken by RGB cameras installed in an ambulance. Figure﻿ 1a shows a conceptual image of the proposed system and Fig. 1b shows an example of two cameras installed in an ambulance. The system uses the camera installed above the head when the patient is lying down with the head-flat position (Fig. 1c) and the camera installed above the feet when the patient is lying down with the head-elevated position (Fig. 1d) to capture video frames of the patient’s face from the front. The proposed system has the potential to introduce quantitative assessment into emergency medicine, enabling more accurate assessment and appropriate transport to medical facilities for suspected stroke patients. Furthermore, the system can be implemented within ambulances using only two RGB cameras for video capture and a computer for video processing.

In order to implement this system, it is essential to develop a method for evaluating CFP from video frames captured with an RGB camera. Here, we propose a simple and robust automatic evaluation system for CFP based on two evaluation indices, namely the symmetry of mouth movement (SMM) and the difference in mouth shape (DMS), respectively. These indices enable the quantitative evaluation of the degree of facial palsy. In this paper, we developed a classification model using two evaluation indices and evaluated its effectiveness using a dataset generated by recording videos for CFP patients and healthy subjects in hospital patient rooms or office rooms. The main contributions of this work are:

1. the proposal of an automatic evaluation system for CFP using RGB cameras installed in an ambulance;
2. the development of two simple evaluation indices, namely SMM and DMS;
3. the development of a classification model that uses these two evaluation indices as features and the evaluation of classification performance for CFP using our dataset including CFP patients and healthy subjects.

## Related work

Previous studies on the automatic evaluation of facial palsy using video or image analysis can be divided into those that focus on central facial palsy (CFP)^9,10^ and those that do not distinguish between CFP and peripheral facial palsy (PFP)^11–25^. For CFP, palsy presents on the lower face, whereas for PFP, palsy presents on the entire face. Although this distinction should be considered in the automatic evaluation of facial palsy in stroke patients, studies on CFP are limited. Previous studies classified CFP, PFP, and healthy people using distances between facial landmarks such as an eye and a mouth corner using machine learning techniques^9,10^. However, such systems were not specially developed for emergency medicine.

Various methods for automatically evaluating peripheral or overall facial palsy have been proposed^11–25^. These methods can be divided into those that use facial landmarks and those that do not.

As a method that does not use facial landmarks, Wang et al.^15^ took images of the head, which was fixed, and analyzed their texture features. Verhoeven et al.^16^ and Codari et al.^17^ analyzed three-dimensional information obtained using a stereophotogrammetry camera. Jiang et al.^18^ used laser speckle contrast imaging. However, it is difficult to use such systems in an ambulance. Some researchers captured images using a general color camera and classified them using a convolutional neural network^19–21^. However, convolutional neural networks are unreliable for clinical use because the basis of their classification cannot be easily understood.

In studies that used facial landmarks, patients were asked to make several expressions, which were captured using a camera; static geometric features were extracted from these images for analysis^22–24^. However, asking a patient to make several expressions during an emergency is impractical. Moreover, methods that use static images, not videos, may fail to identify mild palsy. Monini et al.^25^ performed a video analysis to classify unilateral PFP, focusing on two facial movements (forehead frowning and smiling). They suggested that the landmark-based method (markerless objective method) may be useful for implementing conventional clinical classifications.

## Methods

**Figure 2:**
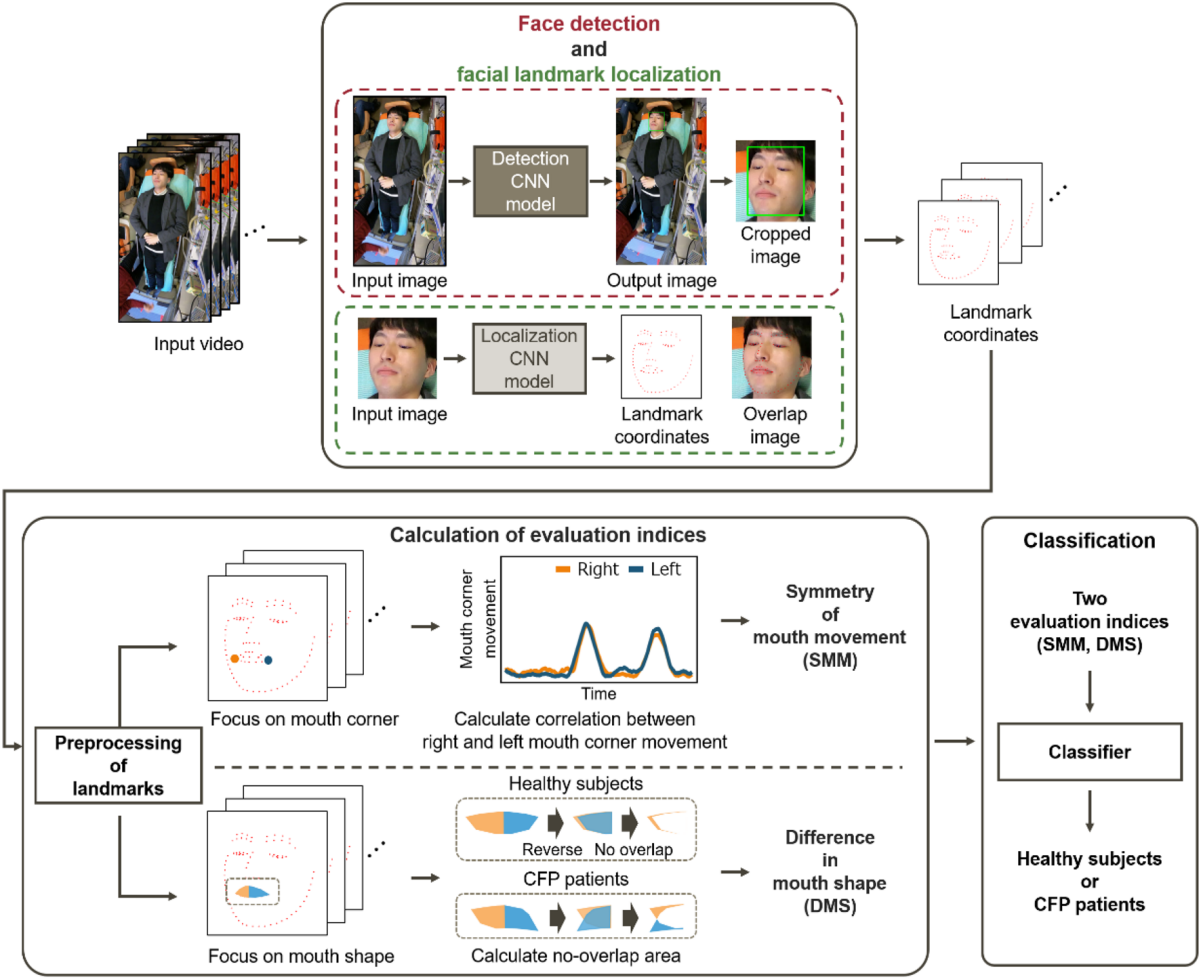
Overview of the proposed automatic evaluation system.

We developed an automatic evaluation system for CFP that uses two evaluation indices calculated from video frames of the face. Figure 2 shows an overview of the proposed system. The system consists of three main steps: (1) face detection and facial landmark localization, (2) calculation of evaluation indices, and (3) classification. The system starts by localizing facial landmarks to calculate the evaluation indices. Then, two evaluation indices, namely SMM and DMS, are calculated from the obtained landmarks (the mouth corner and the mouth contour shape). Finally, the system classifies patients as having or not having CFP. A detailed description of each procedure is presented below.

### Face detection and facial landmark localization

#### Face detection

To detect the facial region in input images, we use FaceBoxes, a face detection deep learning model proposed by Zhang et al.^26^. This model allows sufficiently accurate prediction and the fastest processing among the models we investigated. In this study, instead of training a model from scratch, we use a model trained on WIDER FACE^27^, a face detection benchmark dataset. This trained model is available online at https://github.com/sfzhang15/FaceBoxes.

We apply the model to video frames captured from the front of a subject, obtaining the upper left and lower right coordinates of a bounding box that included the facial region. We crop the facial region from the frame using the estimated coordinates.

#### Facial landmark localization

After face detection, we localize the facial landmarks using a deep learning model proposed by Wang et al.^28^. This model uses a loss function called adaptive wing loss, which adaptively increases loss for foreground pixels and decreases loss for background pixels. Accuracy of landmark localization and sufficient landmarks are essential for calculating the evaluation indices discussed below. Therefore, we selected the model with high accuracy, which is available online, from pre-trained models using a dataset with numerous landmarks. We use a model trained on the Wider Facial Landmarks in the Wild (WFLW)^29^ dataset, which contains 10,000 facial images with 98 annotated landmarks. This trained model is available online at https://github.com/protossw512/AdaptiveWingLoss.

The cropped image is fed to the facial landmark localization model and the coordinates of the 98 facial landmarks defined in the WFLW dataset are generated^29^, as shown in Fig.﻿ 3. We obtain the time series information of the facial landmarks by applying this process to each video frame.

**Figure 3:**
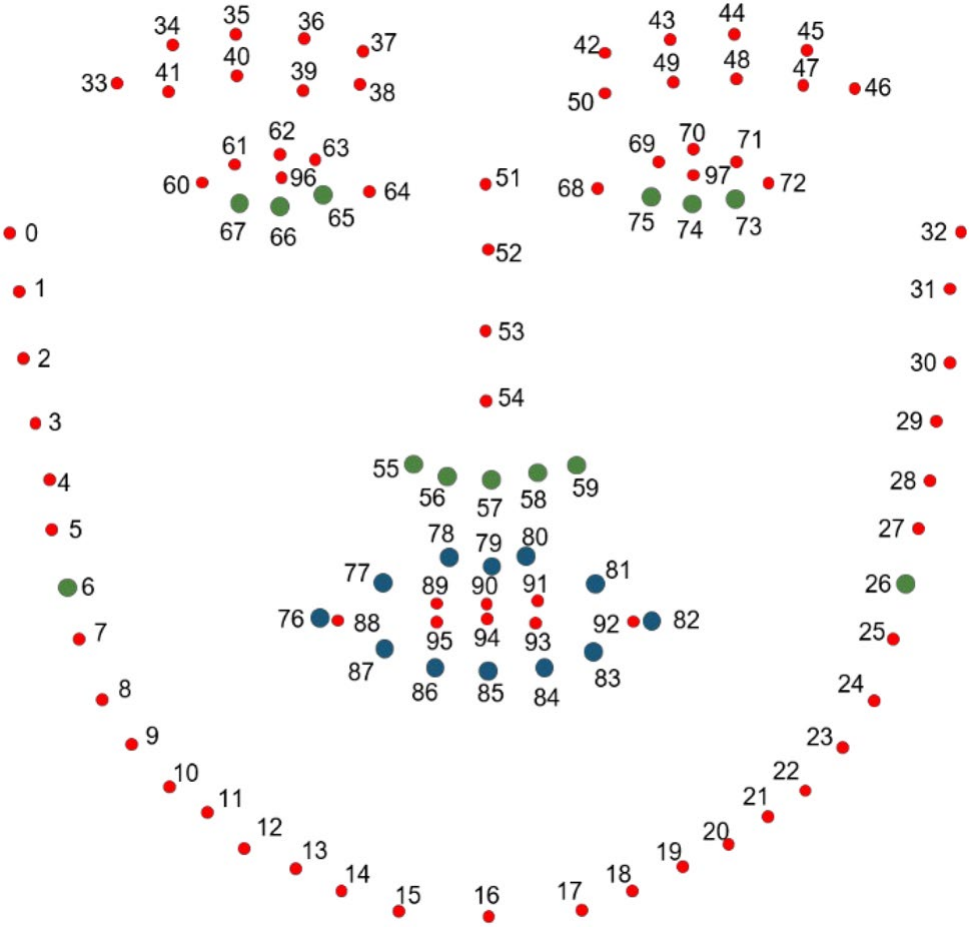
The 98 facial landmarks defined in the WFLW dataset^29^. Blue points are used for the calculation of evaluation indices, green points are used for preprocessing, and red points are not used in our system. This figure was created by the authors based on^29^.

### Calculation of evaluation indices

In general, clinicians instruct patients to “show teeth” and focus on mouth movement and mouth shape when assessing facial palsy in stroke patients. We developed two evaluation indices, namely SMM and DMS, respectively, which reflect the movement of the mouth corner landmarks and the time variation of the area enclosed by the mouth landmarks, respectively. In this section, we first describe the preprocessing procedures for the facial landmarks used to calculate the evaluation indices. Then, we describe the specific calculation methods for SMM and DMS.

#### Preprocessing of landmarks

We need to set the facial midline because the evaluation indices are based on left–right symmetry. Moreover, we need to reduce the influence of the variability of landmark localization in each frame and the variation of face size for an accurate calculation of the evaluation indices.

**Figure 4:**
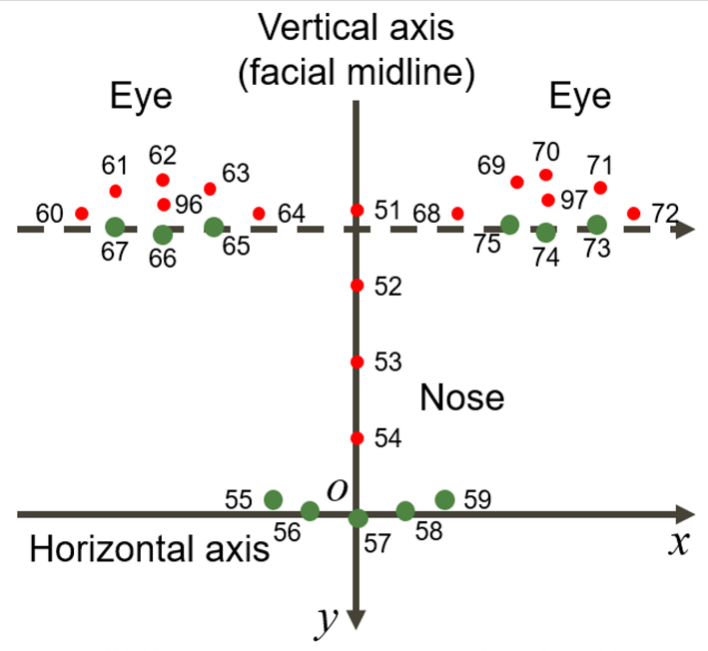
Eye and nose landmarks used to set vertical and horizontal axes. Green points are used for the calculation of the facial midline and red points are not used in our system.

We set the facial midline as follows:

1. Calculate the right and left centers of gravity of three eye-bottom landmarks (shown in Fig. 4, landmark numbers 65–67 and 73–75).
2. Set the center of gravity of five nose-bottom landmarks (shown in Fig. 4, landmark numbers 55–59) as the origin.
3. Calculate the line perpendicular to the segment between the two centers of gravity and through the origin as the facial midline.

Although the facial midline is important for assessing facial symmetry, there is no standard method for calculating the facial midline^30^. Our study uses three eye-bottom landmarks because the eye is less affected by CFP, and the eye bottom is less affected by blepharoptosis compared with the eye top. Furthermore, we use nose landmarks, which can be detected stably, near the mouth to draw a midline in the center of the mouth.

For smoothing, we apply the Gaussian filter. Moreover, we divide the coordinates of all landmarks by the face width calculated from two facial contour landmarks (shown in Fig. 3, landmark numbers 6 and 26), as scale correction to normalize face size in the images.

#### Symmetry of mouth movement (SMM)

One of the symptoms that CFP patients show is asymmetric movement of their mouth. This asymmetry ranges from severe to mild (same as healthy people). Clinicians evaluate severity based on the degree of the symptoms. This symptom is most obvious in the corner of the mouth when patients show their teeth. Therefore, we propose SMM, which reflects the symmetry of the movement of the mouth corner landmarks (shown in Fig. 3, landmark numbers 76 and 82).

**Figure 5:**
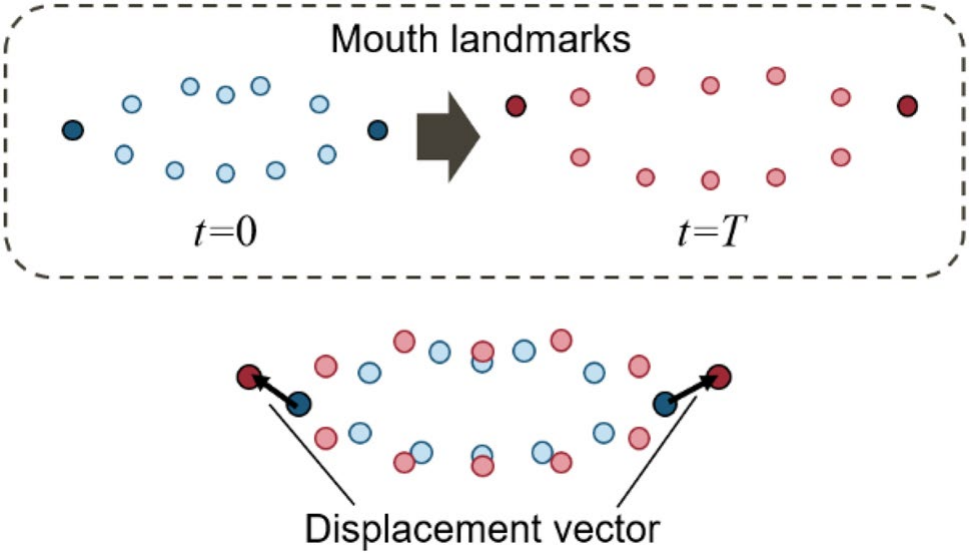
Displacement vectors of mouth corners. Blue points indicate mouth landmarks when *t* = 0 and red points indicate mouth landmarks when *t* = *T.*

To focus on the mouth corner movement, we calculated the displacement vector about two mouth corner landmarks. This displacement vector indicates a landmark movement from *t* to *t* + *T*, as shown in Fig. 5. Here, *t* and *t* + *T* denote frame number and given in integer. *T* represents the time width to calculate the displacement vector and is given the number of frames corresponding to 0.5 s in this study. If the frame rate of image capture is 60 frames per second (fps), *T* equals to 30. This time interval, 0.5 s, was given empirically as a suitable value that can be applied to all subjects.

The calculation of the facial midline affects the direction of the displacement vector and thus we focus on the absolute value of the displacement vector, calculated using Eq. (1).

1 $$\begin{gathered} d_{L,t} = \left\| {{\mathbf{p}}_{t + T}^{(82)} - {\mathbf{p}}_{t}^{(82)} } \right\|, \hfill \\ d_{R,t} = \left\| {{\mathbf{p}}_{t + T}^{(76)} - {\mathbf{p}}_{t}^{(76)} } \right\|, \hfill \\ \end{gathered}$$

where **p**_*t*_^(*n*)^ is the position vector (*x, y*) of landmark *n* at *t*, *d*_*R,t*_ is the absolute value of the displacement vector of the right mouth corner (landmark number 76) at *t*, *d*_*L,t*_ is the absolute value of the displacement vector of the left mouth corner (landmark number 82) at *t*, and ||·|| is the Euclidean norm*.*

SMM reflects the correlation between the movement of the left and right mouth corners. It is defined as follows:

2 $${\text{SMM}} = \frac{{\sum\limits_{i = 1}^{N - T} {d_{R,i} d_{L,i} } }}{{\sqrt {\sum\limits_{i = 1}^{N - T} {d_{R,i}^{2} } } \sqrt {\sum\limits_{i = 1}^{N - T} {d_{L,i}^{2} } } }},$$

where* N* is the number of frames. A value close to 1 indicates symmetric movement and that close to 0 or negative indicates asymmetric movement.

#### Difference in mouth shape (DMS)

Another symptom that CFP patients show is a drooping mouth corner. This symptom is caused by the droop of the whole affected side due to a flaccidity of the facial muscles. This droop ranges from severe (complete droop) to mild (minimal asymmetry). Clinicians evaluate severity based on the degree of the symptoms. Therefore, we propose DMS, which reflects the difference in mouth shape caused by drooping mouth corners.

**Figure 6:**
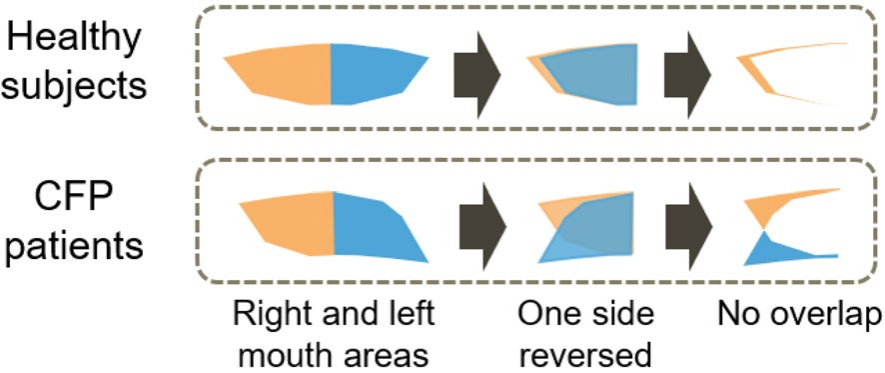
Overview of the calculation for DMS. Orange and blue areas indicate the right and left mouth areas enclosed by the mouth contour landmarks, respectively.

**Figure 7:**
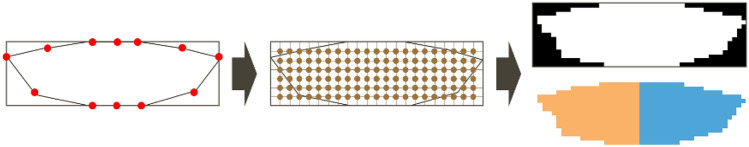
Calculation of mouth area. The rectangle area width is divided into 200 grids, and the height is divided to ensure that the grids of squares.

An overview of the DMS calculation is shown in Fig. 6. First, we calculate the right and left areas enclosed by the mouth contour landmarks and facial midline, as shown in ﻿Fig. 7. We calculate the area as follows:

1. Divide the rectangle area circumscribed at the mouth contour landmarks into square grids.
2. Calculate the straight line that connects neighboring landmarks.
3. Extract the inside of the mouth contour.

We divided the rectangle width into 200 grids, and the height was divided to ensure that the grids of squares. Then, we calculate the no-overlap area when folding one side based on the facial midline. When the subject is healthy, the no-overlap area is small. In contrast, when the subject has facial palsy, the no-overlap area is large due to the droop of the mouth corner. We define DMS as the average of the no-overlap area in the time direction as follows:

3 $${\text{DMS}} = \frac{1}{N}\sum\limits_{i = 1}^{N} {\frac{{\left| {R_{i} \oplus L_{i} } \right|}}{{\left| {R_{i} } \right| + \left| {L_{i} } \right|}}}$$

where *R*,* L* is a set consisting of the grid points enclosed by the midline and the mouth contour line formed by connecting the two closest landmarks to each other, ⊕ is the exclusive disjunction of the set, and *N* is the number of frames. |*X*| represents the number of elements of the set *X*. A value close to 0 indicates a symmetric shape and that close to 1 indicates an asymmetric shape.

### Classification

Finally, the system classifies subjects into CFP patients and healthy subjects using a classifier with the two evaluation indices. Logistic regression is used to perform the classification. We selected this simple classifier to emphasize the effectiveness of the two evaluation indices.

## Experiments

We evaluated the performance of the two evaluation indices and the proposed automatic evaluation system using experiments. In this section, we introduce the dataset used in the experiments, statistical analysis methods for the two evaluation indices, and classification performance evaluation methods for the proposed system. All experiments were implemented in Python 3.8.5 on a computer with an Intel Core i7-9700K CPU (3.60 GHz, 8 cores) and 64 GB of RAM.

### Dataset

Eighteen CFP patients (ten with mild palsy and eight with severe palsy) in their fifties to nineties (Mean ± SD: 73.8 ± 11.3) participated in this study. Patients were under treatment at Chiba University Hospital and Chiba Medical Center. The clinician determined the severity of these patients based on the National Institutes of Health Stroke Scale (NIHSS). The protocol was approved by the Ethics Review Board of Chiba University (approval number: clinical 2259) and informed consent was obtained from all patients before their inclusion. Furthermore, 20 healthy volunteers in their twenties to fifties (Mean ± SD: 27.2 ± 9.0) participated in this study. All research were performed in accordance with the Ethical Guidelines for Medical and Health Research Involving Human Subjects in Japan.

We placed a GoPro HERO8 Black camera (GoPro, Inc., San Mateo, CA) in front of the patients or healthy subjects and recorded facial movies under the condition that they were sitting on a chair or lying on a bed in hospital patient rooms or office rooms. We instructed them to display neutral expressions, show teeth, and then close their mouth a few seconds later. The videos were acquired at 30 or 60 fps at a resolution of 1920 × 640 pixels.

### Statistical analysis

We performed the Mann–Whitney U test, a nonparametric method that does not assume a normal distribution or large sample size, to evaluate the difference in each evaluation index (SMM and DMS) between the healthy subject group (20 subjects) and the CFP patient group (18 patients). Furthermore, we qualitatively evaluated values for each evaluation index between healthy subjects, mild patients, and severe patients to analyze the details of patients’ data.

### Classification performance evaluation

We classified our dataset as healthy subjects and CFP patients using the proposed system and evaluated the classification performance. Since the amount of data was small, leave-one-out cross-validation was used for evaluation. Specifically, we split the dataset (with N samples) into N−1 samples and one sample and used the N−1 samples as training data and the one sample as testing data. Furthermore, to evaluate classification performance, we used the receiver operating characteristic (ROC) curve, where the vertical axis is the true positive rate and the horizontal axis is the false positive rate.

As an evaluation index, we used the area under the curve (AUC), specifically the area under the ROC curve. A value of AUC close to 1 indicates high classification performance and that close to 0.5 indicates random classification. Furthermore, the Youden index was used to determine the optimal threshold (i.e., the maximum point of true positive rate − false positive rate). The accuracy, sensitivity, and specificity were then calculated.

## Results

### Statistical analysis results

**Figure 8:**
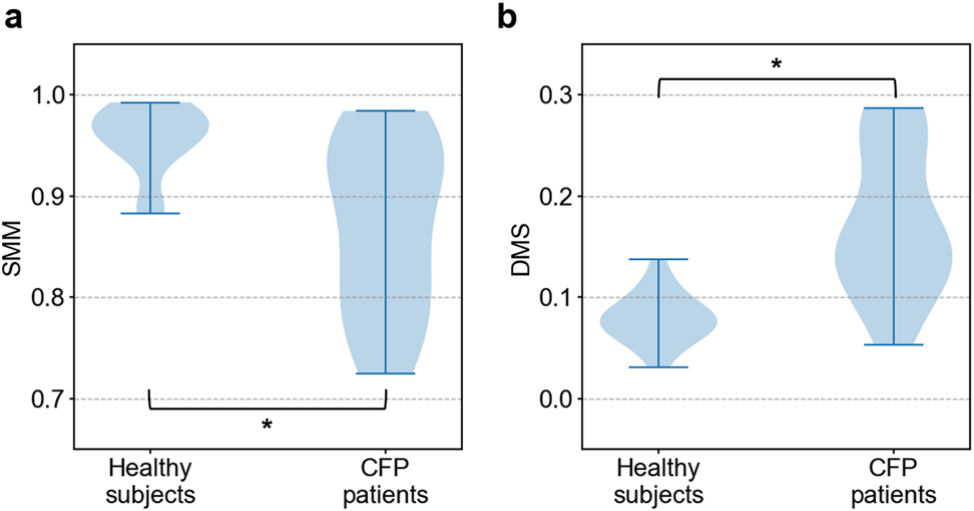
Quantitative results for healthy subjects and CFP patients obtained using **a** SMM and **b** DMS. Asterisk indicates statistically significant differences between healthy subjects and CFP patients (significance level was set to 5%).

**Figure 9:**
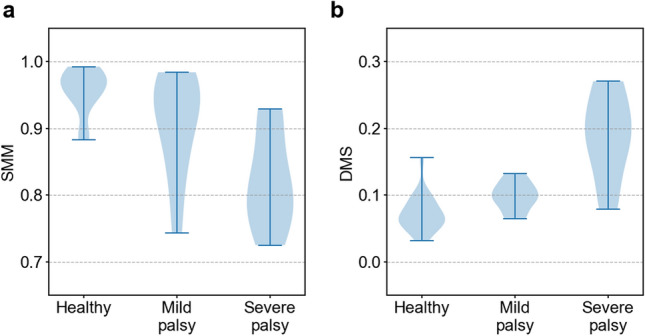
Relationship between evaluation indices and severity. Results for **a** SMM and **b** DMS.

Figure 8 shows violin plots of SMM and DMS for the healthy subject group and the CFP patient group. These violin plots indicate that there are differences in the values of each evaluation index between the two groups. The U test results for SMM and DMS show significant differences (significance level was set to 5%) between healthy subjects and CFP patients for each index (SMM: *p* = 4.27 × 10^–4^, DMS: *p* = 8.42 × 10^–5^). Figure 9 shows the relationship between the two evaluation indices and palsy severity based on the violin plots. Table 1 shows the severity and evaluation index values for each subject. For SMM, half of the mild palsy patients and some severe palsy patients had values within the range of healthy subjects, as shown in Fig. 9a. For DMS, all mild palsy patients and some severe palsy patients had values within the range of healthy subjects, as shown in Fig. 9b.

**Table 1:** Severity and evaluation index values for each subject.

Patient	Sex	SMM	DMS	Patient	Sex	SMM	DMS
Healthy-1	M	0.88	0.07	Healthy-20	M	0.99	0.06
Healthy-2	M	0.94	0.07	Mild-1	F	0.74	0.12
Healthy-3	M	0.94	0.10	Mild-2	F	0.92	0.09
Healthy-4	M	0.97	0.06	Mild-3	F	0.89	0.13
Healthy-5	F	0.98	0.09	Mild-4	M	0.95	0.12
Healthy-6	M	0.96	0.16	Mild-5	F	0.97	0.06
Healthy-7	M	0.98	0.03	Mild-6	M	0.98	0.10
Healthy-8	M	0.99	0.05	Mild-7	F	0.82	0.10
Healthy-9	M	0.95	0.04	Mild-8	F	0.96	0.10
Healthy-10	M	0.96	0.07	Mild-9	M	0.95	0.10
Healthy-11	F	0.97	0.06	Mild-10	F	0.86	0.07
Healthy-12	M	0.95	0.09	Severe-1	M	0.73	0.27
Healthy-13	F	0.99	0.10	Severe-2	M	0.82	0.18
Healthy-14	M	0.98	0.06	Severe-3	M	0.93	0.19
Healthy-15	M	0.97	0.06	Severe-4	F	0.93	0.19
Healthy-16	M	0.96	0.06	Severe-5	M	0.79	0.27
Healthy-17	M	0.97	0.09	Severe-6	M	0.78	0.08
Healthy-18	M	0.88	0.07	Severe-7	M	0.76	0.12
Healthy-19	M	0.89	0.05	Severe-8	F	0.85	0.22

### Classification performance evaluation results

**Figure 10:**
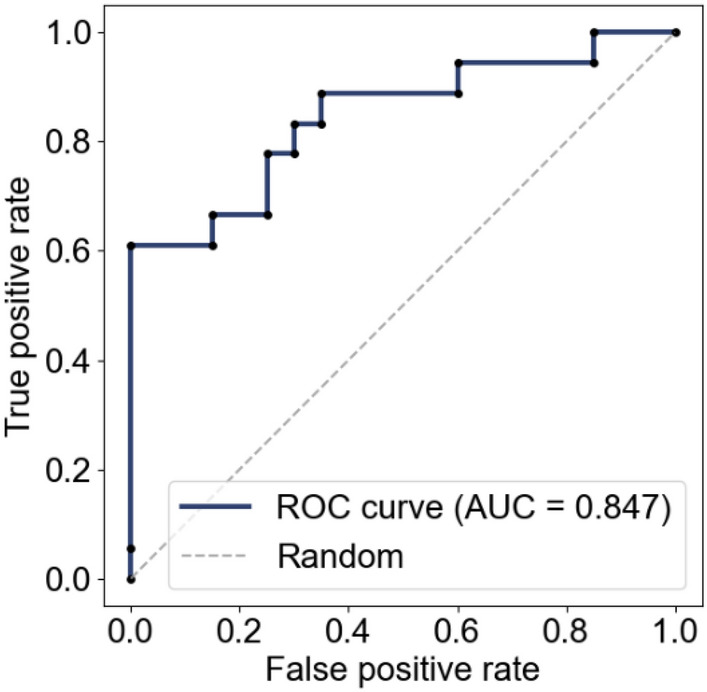
ROC curve of patient classification using logistic regression.

Figure 10 shows the ROC curve for patient classification obtained using logistic regression. The classification achieved an AUC of 0.847. Table 2 summarizes the classification results using the optimal cutoff point calculated using the Youden index (0.61). A sensitivity of 61.0%, a specificity of 100.0%, and an accuracy of 81.6% were obtained. The two evaluation indices had significant differences between healthy subjects and patients, who were thus classified with high accuracy.

**Table 2:** Confusion matrix for logistic regression classifier.

	Predicted CFP patients	Predicted healthy subjects
Actual CFP patients	11	7
Actual healthy subjects	0	20

## Discussion

The proposed automatic evaluation system can accurately classify CFP patients using two evaluation indices (SMM and DMS) and logistic regression. This simple classifier was used in this study to emphasize the effectiveness of the two evaluation indices. SMM and DMS were developed considering important clinical findings. They are thus not only explanatory variables of a classifier but also indicators of the degree asymmetry of mouth movement and mouth shape, respectively. CFP is characterized by palsy shown on the lower face. To the best of our knowledge, this is the first study to automatically evaluate CFP using only information about the lower face. The experimental results demonstrate that the proposed method achieves high classification accuracy.

We measured the processing time for detecting face images, localizing facial landmarks, and calculating evaluation indices from the localized landmarks. First, our measurement test showed that the processing time from face detection to landmark localization is 0.096 s per frame. In our dataset, each video consists of staying neutral expression (a few seconds), smiling (a few seconds), and returning to a neural expression (a few seconds), with the total video duration not exceeding ten seconds. Thus, processing a ten-second video at 30 fps (300 frames) requires approximately 30 s. Second, calculating the evaluation indices from the localized landmarks requires approximately 35 s. In total, the entire processing pipeline takes about 65 s. In the future, it will be important to reduce the processing time of the entire pipeline and to develop a system that provides immediate feedback.

Our dataset shows a huge age difference between the patient and the healthy subject groups. It is acknowledged that aging may cause a weakening of the movement at the corners of the mouth, we recognize that in the evaluation of facial palsy, the difference in the movement of the right and left corners of the mouth is more crucial than the age difference. Therefore, we believe that the datasets used in this paper are reasonable for evaluating the effectiveness of the proposed method. However, to more convincingly demonstrate the effectiveness of the proposed method, it would be desirable to match the age of the patient and healthy subject groups. Furthermore, the dataset used to verify the effectiveness of the proposed method does not consist of videos captured inside an ambulance. Instead, the dataset was generated by recording videos in hospital patient rooms or office rooms. In future work, we will collect additional data from CFP patients and healthy subjects captured within ambulances and expand our datasets.

The limitations of the proposed system are as follows. First, for some subjects, the system failed to localize facial landmarks correctly and could not capture the facial contours and midline. Consequently, DMS could not adequately reflect the asymmetric mouth shape. Landmark localization error may explain why the DMS values for many mild palsy patients and some severe palsy patients (Severe-6 and Severe-7) were similar to those for healthy subjects. In contrast, the SMM values, which were calculated using only the landmarks at the left and right corners of the mouth, were not significantly affected by localization error. These results suggest that common landmark localization models cannot accurately localize landmarks for facial palsy when trained using data from only healthy subjects. Some studies^31,32^ suggested that accuracy can be improved using a dataset that includes facial palsy patients. It is thus expected that training with large datasets that include data from facial palsy patients will improve the validity of the assessments with landmarks.

Second, some cases could not be handled by the two evaluation indices. We qualitatively confirmed that the mouth movement of some patients (Mild-2, Mild-4, Mild-5, Mild-6, Mild-8, Mild-9, Severe-3, and Severe-4) was similar to that of the healthy subjects and the mouth shape of some patients (Mild-1, Mild-5, Mild-6, Mild-7, and Mild-9) was symmetric or mildly asymmetric. As a result, the evaluation index values for these patients were similar to those of the healthy subjects. In addition, the mouth movement of Healthy-19 and the mouth shape of Healthy-6 were asymmetric. Our findings suggest that landmark-based evaluation indices alone cannot handle these cases. Therefore, for future work, we need to consider additional approaches that are not solely dependent on landmarks. This may include exploring evaluation indices based on texture features or leveraging cameras capable of capturing additional information, such as RGB-D or thermal cameras. In addition, the proposed system cannot correctly evaluate complete facial palsy patients. The movement of the mouth corner of complete facial palsy patients is non-existent or very small and thus cannot be properly measured. In the future, the proposed system requires a function to identify complete facial palsy patients considering the existence of the movement.

Third, some subjects failed to correctly perform the “show teeth” motion. The mouth movement of Healthy-1 was smaller than that of the other healthy subjects; the SMM value for this subject was influenced by landmark localization error between frames (whose amplitude was similar to that of the mouth movement). Furthermore, Healthy-18 closed the mouth gradually by slightly straining the mouth muscles before the instruction to close the mouth, resulting in asymmetric mouth movement. Thus, we need to improve the instructions regarding mouth movement. Volk et al.^33^ reported that the reliability of an evaluation conducted using video instructions was excellent. For example, installing monitors on the ceiling of an ambulance and asking patients to perform movements shown in the instruction video may reduce instruction errors.

## Conclusion

We proposed a simple automatic evaluation system for CFP based on two evaluation indices, namely SMM and DMS, which reflect the movement of the mouth corner landmarks and the time variation of the area enclosed by the mouth landmarks, respectively. The values of each index were significantly different between healthy subjects and CFP patients. Our system thus accurately classified subjects (AUC was 0.847). However, limitations include insufficient accuracy of landmark localization and somewhat unreliable instructions for performing the “show teeth” motion. In future work, we will improve landmark localization accuracy and consider practical problems such as improving the instructions for patients.

## Data Availability

The datasets used and analyzed during our study are not publicly available because they contain facial information of the subjects but are available from the corresponding author upon reasonable request.
